# Tumor suppressive *microRNA-138* contributes to cell migration and invasion through its targeting of vimentin in renal cell carcinoma

**DOI:** 10.3892/ijo.2012.1543

**Published:** 2012-07-03

**Authors:** TAKESHI YAMASAKI, NAOHIKO SEKI, YASUTOSHI YAMADA, HIROFUMI YOSHINO, HIDEO HIDAKA, TAKESHI CHIYOMARU, NIJIRO NOHATA, TAKASHI KINOSHITA, MASAYUKI NAKAGAWA, HIDEKI ENOKIDA

**Affiliations:** 1 Department of Urology, Graduate School of Medical and Dental Sciences, Kagoshima University, Kagoshima;; 2 Department of Functional Genomics, Graduate School of Medicine, Chiba University, Chiba, Japan

**Keywords:** microRNA, *miR-138*, *vimentin*, renal cell carcinoma

## Abstract

Many studies have recently suggested that microRNAs (miRNAs) contribute to the development of various types of human cancers as well as to their invasive and metastatic capacities. Previously, our miRNA expression signature of renal cell carcinoma (RCC) revealed that *microRNA-138* (*miR-138*) was significantly reduced in cancer cells. The aim of the present study was to investigate the functional significance of *miR-138* and to identify its target genes in RCC cells. Restoration of mature *miR-138* in two RCC cell lines (A498 and 786-O) caused changes in the bleb-like cell morphology, characteristics of the epithelial-mesenchymal transition (EMT). Restoration also significantly inhibited migration and invasion in the two RCC cell lines, suggesting that *miR-138* functions as a tumor suppressor. Genome-wide gene expression analysis (*miR-138* transfectants and RCC clinical specimens) and TargetScan database studies showed that vimentin (*VIM*) is a promising candidate target gene of *miR-138*. It is well known that VIM is one of the most widely expressed mammalian intermediate filament proteins. Recent studies showed that VIM functions in cell adhesion, migration, survival and cell signaling processes via dynamic assembly/disassembly in cancer cells. We focused on VIM and investigated whether VIM was regulated by tumor suppressive *miR-138* and contributed to cancer cell migration and invasion in RCC cells. Restoration of *miR-138* in RCC cell lines suppressed VIM expression at both the mRNA and protein levels. Silencing studies of VIM in RCC cell lines demonstrated significant inhibition of cell migration and invasion activities in si-VIM transfectants. In clinical specimens of RCC, the expression levels of *VIM* were significantly upregulated in cancer tissues compared to adjacent non-cancerous tissues. Furthermore, immunohistochemistry showed that VIM expression levels in RCC specimens were significantly higher than those in normal renal tissues. These data suggest that VIM may function as an oncogene and is regulated by tumor suppressive *miR-138*. The existence of a tumor suppressive *miR-138*-mediated oncogenic pathway provides new insights into the potential mechanisms of RCC oncogenesis and metastasis.

## Introduction

Renal cell carcinoma (RCC) is the most common neoplasm of the adult kidney. In this disease, cancer cells form in the tubules of the kidney and approximately 80% of RCC patients are diagnosed with the clear cell RCC subtype (1). Up to 30% of RCC patients present at advanced stages, and approximately 40% of patients who undergo curative surgical resection experience recurrence during subsequent follow-up (2,3). The five-year survival rate of advanced RCC is 5–10% (4). RCC is resistant to radiotherapy and chemotherapy (5,6). Targeted therapies such as sunitinib, sorafenib, everolimus and temsirolimus have been developed and have been used widely in first- and second-line treatments, extending the period of progression-free-survival (7,8). However, these treatments are insufficient for patients who have developed relapse or metastasis. Therefore, increased understanding of the molecular mechanisms of RCC progression and metastasis is needed using the latest approaches to genomic analysis.

RNA can be divided into two categories: protein coding RNA and non-coding RNA (ncRNA). It is important to examine the functions of ncRNAs and their association with human disease, including cancer. microRNAs (miRNAs) are endogenous small ncRNA molecules (19–22 bases in length) that regulate protein coding gene expression by repressing translation or cleaving RNA transcripts in a sequence-specific manner (9). A growing body of evidence suggests that miRNAs are aberrantly expressed in many human cancers, and that they play significant roles in their initiation, development, and metastasis (10). Some highly expressed miRNAs could function as oncogenes by repressing tumor suppressors, whereas low level miRNAs could function as tumor suppressors by negatively regulating oncogenes (11).

We previously identified many tumor suppressive miRNAs based on our miRNA expression signatures of various types of cancer, such as RCC, bladder cancer, prostate cancer, maxillary sinus squamous cell carcinoma and hypopharyngeal squamous cell carcinoma (12–17). In oncogenic pathways, normal regulatory mechanisms are disrupted by aberrant expression of tumor suppressive or oncogenic miRNAs. Therefore, identification of miRNA-regulated pathways is important for further development in human cancer research. Thus, we have been investigating how tumor suppressive miRNA regulates novel cancer pathways. For example, the *miR-1/miR-133a* cluster regulates several oncogenic genes, including transgelin-2 (*TAGLN2*), prothymosin α (*PTMA*) and purine nucleoside phosphorylase (*PNP*) (14,15,18).

More recently, we constructed a miRNA expression signature of RCC clinical specimens and successfully identified tumor suppressive *miR-1285* targeting transglutaminase 2 (*TGM2*) (12). Among the signatures, several miRNAs were significantly downregulated in RCC specimens as promising candidate of tumor suppressors. In this study, we focused on *miR-138*. This miRNA was downregulated in our previous signature, and downregulation of *miR-138* has been observed in several malignancies, including anaplastic thyroid carcinoma (19) and lung cancer (20).

The aim of the study was to investigate the functional significance of *miR-138* and identify its target genes in RCC cells. To identify *miR-138*-regulated cancer pathways, we undertook both a genome-wide gene expression analysis (*miR-138* transfectants and RCC clinical specimens) and an *in silico* study. The results showed that vimentin (*VIM*) was a promising candidate target gene of *miR-138*. It is well known that VIM is one of the most widely expressed mammalian intermediate filament proteins. Studies have shown that VIM functions in cell adhesion, migration, survival, and cell signaling processes via dynamic assembly/disassembly in cancer cells (21). The existence of a tumor suppressive *miR-138*-mediated cancer pathway provides new insights into the potential mechanisms of RCC oncogenesis and metastasis.

## Materials and methods

### Clinical specimens

A total of 33 pairs of clear cell renal cell carcinoma (ccRCC) and adjacent non-cancerous specimens were collected from patients who had undergone radical nephrectomies at Kagoshima University Hospital. The samples were processed and stored in RNAlater (Qiagen, Valencia, CA, USA) at −20°C until RNA extraction. The patient information is summarized in Table I. These samples were staged according to the American Joint Committee on Cancer-Union Internationale Contre le Cancer (UICC) tumour-node-metastasis classification and histologically graded (22). Our study was approved by the Bioethics Committee of Kagoshima University; written prior informed consent and approval were given by the patients.

### Cell culture and RNA extraction

We used two human RCC cell lines, A498 and 786-O, obtained from the American Type Culture Collection (Manassas, VA, USA). The cell lines were incubated in RPMI-1640 medium supplemented with 10% fetal bovine serum (FBS) and maintained in a humidified incubator (5% CO_2_) at 37°C. Total-RNA was extracted, as previously described (12).

### Quantitative real-time RT-PCR

TaqMan probes and primers for *VIM* (P/N: Hs00185584_m1: Applied Biosystems) were assay-on-demand gene expression products. All reactions were performed in duplicate, and a negative control lacking cDNA was included. We followed the manufacturer’s protocol for PCR conditions. Stem-loop RT-PCR (TaqMan MicroRNA Assays; P/N: 002284 for miR-138; Applied Biosystems) was used to quantitate miRNAs according to the earlier published conditions (23). To normalize the data for quantification of *VIM* mRNA and the miRNAs, we used *human GUSB* (P/N: Hs99999908_m1; Applied Biosystems) and *RNU6B* (P/N: 001973; Applied Biosystems), respectively, and we used the ΔΔCt method to calculate the fold-change. As a control RNA, we used Premium total-RNA from normal human kidney (AM 7976; Applied Biosystems).

### Mature miRNA and siRNA transfection

As described elsewhere (23), the RCC cell lines were transfected with Lipofectamine™ RNAiMAX transfection reagent (Invitrogen, Carlsbad, CA, USA) and Opti-MEM™ (Invitrogen) with 10 nM mature miRNA molecules. Pre-miR™ (Applied Biosystems) and negative-control miRNA (Applied Biosystems) were used in the gain-of-function experiments, whereas *VIM* siRNA (Cat nos. SASI_ Hs01_00044033 and SASI_HS01_00044036, Sigma-Aldrich, St. Louis, MO, USA) and negative control siRNA (D-001810-10; Thermo Fisher Scientific, Waltham, MA, USA) were used in the loss-of-function experiments. Cells were seeded in 10-cm dishes for protein extraction (8×10^5^ cells per dish), 6-well plates for wound healing assays (20×10^4^ cells per well), in 24-well plates for the mRNA extraction and Matrigel invasion assays (5×10^4^ cells per well) and in 96-well plates for the XTT assays (3,000 cells per well).

### Cell morphology

Cells were transfected with *miR-138* and si-*VIM* for 72 h and were then examined by an inverted microscope (CK2-BIP2, Olympus).

### Cell proliferation, migration and invasion assays

Cell proliferation was determined using an XTT assay (Roche Applied Science, Tokyo, Japan) that was performed according to the manufacturer’s instructions. Cell migration activity was evaluated with a wound healing assay. Cells were plated in 6-well dishes and the cell monolayer was scraped using a P-20 micropipette tip. The initial gap length (0 h) and the residual gap length 24 h after wounding were calculated from photomicrographs. A cell invasion assay was carried out using modified Boyden Chambers consisting of Transwell-precoated Matrigel membrane filter inserts with 8-mm pores in 24-well tissue cultures plates (BD Bioscience, Bedford, MA, USA). Minimum essential medium containing 10% FBS in the lower chamber served as the chemoattractant as described previously (24). All experiments were performed in triplicate.

### Screening of miR-138-regulated genes by microarray

Oligomicroarray Human 60K (Agilent) was used for expression signature in *miR-138*-transfected A498 cells in comparison with the miR-negative control transfectant, as previously described (23). Briefly, hybridization and washing steps were performed in accordance with the manufacturer’s instructions. The arrays were scanned using a Packard GSI Lumonics ScanArray 4000 (PerkinElmer, Boston, MA, USA). The data obtained were analyzed with DNASIS array software (Hitachi Software Engineering, Tokyo, Japan) that converted the signal intensity. Data from each microarray study were normalized by global normalization.

### Expression signature of RCC clinical specimens by microarray

Oligo-microarray Human 60K (Agilent) was used for expression signature in 5 pairs of RCC clinical specimens compared with adjacent non-cancerous tissues. Their age ranged from 42 to 77 years; 3 were G1 and 2 were G2 in their tumor grading; and all were pT1N0M0 tumors.

### Western blot analysis

After three days of transfection, protein lysates (40 *μ*g) were separated by NuPAGE on 4–12% bis-tris gels (Invitrogen) and transferred onto polyvinylidene fluoride membranes. Immunoblotting was done with diluted (1:500) polyclonal VIM antibody (HPA001762; Sigma-Aldrich) and GAPDH antibody (MAB374; Chemicon, Temecula, CA, USA). The membrane was washed and then incubated with goat anti-rabbit IgG (H+L)-HRP conjugate (Bio-Rad, Hercules, CA, USA). Specific complexes were visualized with an echochemiluminescence (ECL) detection system (GE Healthcare, Little Chalfont, UK), and the expression levels of these genes were evaluated by ImageJ software (ver. 1.43; http://rsbweb.nih.gov/ij/index.html).

### Immunohistochemistry

A tissue microarray of 67 RCC samples and 10 normal kidney samples was obtained from US Biomax Inc. (KD806; Rockville, MD, USA). Detailed information on all tumor specimens can be found at http://www.biomax.us/index.php. Patient characteristics are summarized in Table III. Immunostaining was done on the tissue microarray following the manufacturer’s protocol by UltraVision Detection System (Thermo Scientific). The primary rabbit polyclonal antibodies against VIM (Sigma-Aldrich) were diluted 1:500. The slides were treated with biotinylated goat anti-rabbit. Diaminobenzidine hydrogen peroxidase was the chromogen, and the counterstaining was done with 0.5% hematoxylin. Immunostaining was evaluated according to a scoring method described previously (14). Each case was scored on the basis of the intensity and area of staining. The intensity of staining was graded on the following scale: 0, no staining; 1+, mild staining; 2+, 30–60% stained positive; 3+, >60% stained positive. A combined staining score (intensity + extent) of <2 was low expression, a score between 3 and 4 was moderate expression, and a score between 5 and 6 was high expression.

### Statistical analysis

The relationships between two variables and numerical values were analyzed using the Mann-Whitney U test, and the relationship between three variables and the numerical values was analyzed using the Bonferroni-adjusted Mann-Whitney U test. Expert Stat View analysis software (ver. 4; SAS institute Inc., Cary, NC, USA) was used in both analyses. In the comparison of three variables, a non-adjusted statistical level of significance of P<0.05 corresponded to the Bonferroni-adjusted level of P<0.0167.

## Results

### Effect of miR-138 transfection on cell proliferation, migration, and invasion activity of RCC cell lines

**Figure 1:**
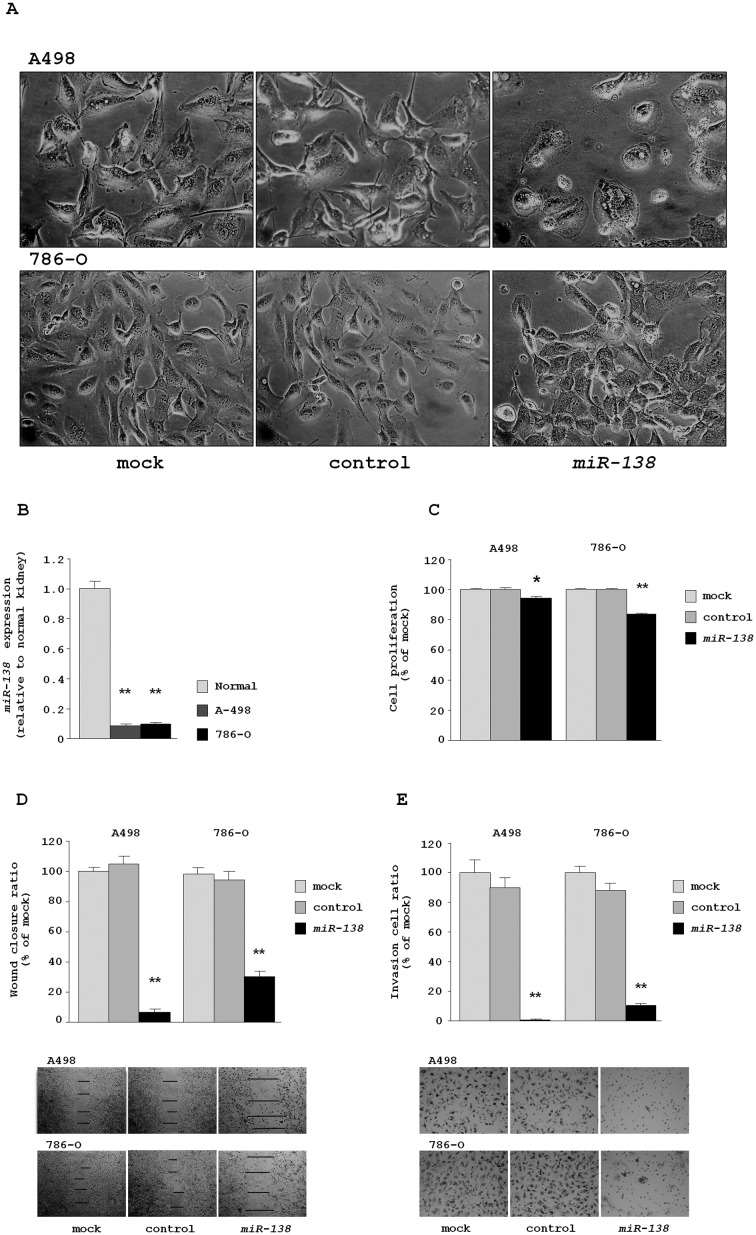
Effect of *miR-138* transfection on RCC cell lines. (A) The change of morphology of *miR-138* transfectants. A498 and 786-O cells were transfected with *miR-138* for 72 h and were then examined by an inverted microscope. (B) *miR-138* expression in A498 and 786-O cell lines and in normal kidney. *miR-138* expression levels in A498 and 786-O were significantly lower than those in normal human kidney RNA. *RNU6B* was used as an internal control. (C–E) Effect of *miR-138* transfection of A498 and 786-O cells. (C) Cell proliferation determined by the XTT assay; (D) cell migration activity determined by wound healing assay; and (E) cell invasion activity determined by the Matrigel invasion assay. ^*^P<0.001, ^**^P<0.0001.

In this study, we firstly observed that restoration of *miR-138* in RCC cell lines (A498 and 786-O) changed the bleb-like cell morphology characteristic of the epithelial-mesenchymal transition (EMT) (Fig. 1A). A morphological change of cancer cells by miRNA transfection is an important discovery and it suggested that *miR-138* functions as a tumor suppressor in RCC cells. To explore that possibility, the following experiments were conducted.

We evaluated the expression levels of *miR-138* in two RCC cell lines, A498 and 786-O. RNA was extracted and miRNA expression levels of *miR-138* were determined by real-time RT-PCR. The expression levels of *miR-138* were significantly lower in both RCC cell lines compared with normal kidney RNA (relative to normal kidney RNA, 0.090±0.008 and 0.102±0.009, respectively) (Fig. 1B).

The XTT assay revealed that cell proliferation was significantly inhibited in *miR-138* transfectants in comparison with the transfectant reagent only (mock) and the miR-control transfectants. The percentages of cell proliferation for A498 were 94.6±0.9, 100.0±0.8 and 100.0±1.0, respectively, each P=0.0008. For 786-O, the percentages were 83.5±1.1, 100.0±0.4 and 100.3±0.6, respectively, P<0.0001 (Fig. 1C).

The wound healing assay demonstrated that significant inhibition of cell migration occurred in the *miR-138* transfectants in comparison with mock and the miR-control transfectants. The percentages of wound closure for A498 were 6.5±2.3, 100.0±2.7 and 104.8±4.9, respectively, each P<0.0001. For 786-O, the percentages were 30.7±3.8, 100.0±4.4 and 95.9±5.4, respectively, each P<0.0001 (Fig. 1D).

The Matrigel invasion assay demonstrated that the number of invading cells significantly decreased in the *miR-138*-transfectants in comparison with mock and the miR-control transfectants. The percentages of cell invasion for A498 were 0.9±0.4, 100.0±8.6 and 83.1±7.4, respectively, each P<0.0001. For 786-O, the values were 10.9±1.1, 100.0±4.4 and 75.3±6.2, respectively, each P<0.0001 (Fig. 1E).

### miR-138 regulation of molecular targets assessed by genome-wide gene expression analysis

To confirm that *miR-138* regulated molecular targets in RCC cells, we performed genome-wide gene expression analysis using *miR-138* transfectants compared with miRNA-control transfectants in A498 cells. A total of 99 genes were downregulated in *miR-138* transfectants. Among them, 24 genes had putative target site(s) in their 3′ untranslated region (3′UTR) according to the TargetScan miRNA program (Table II).

**Figure 2:**
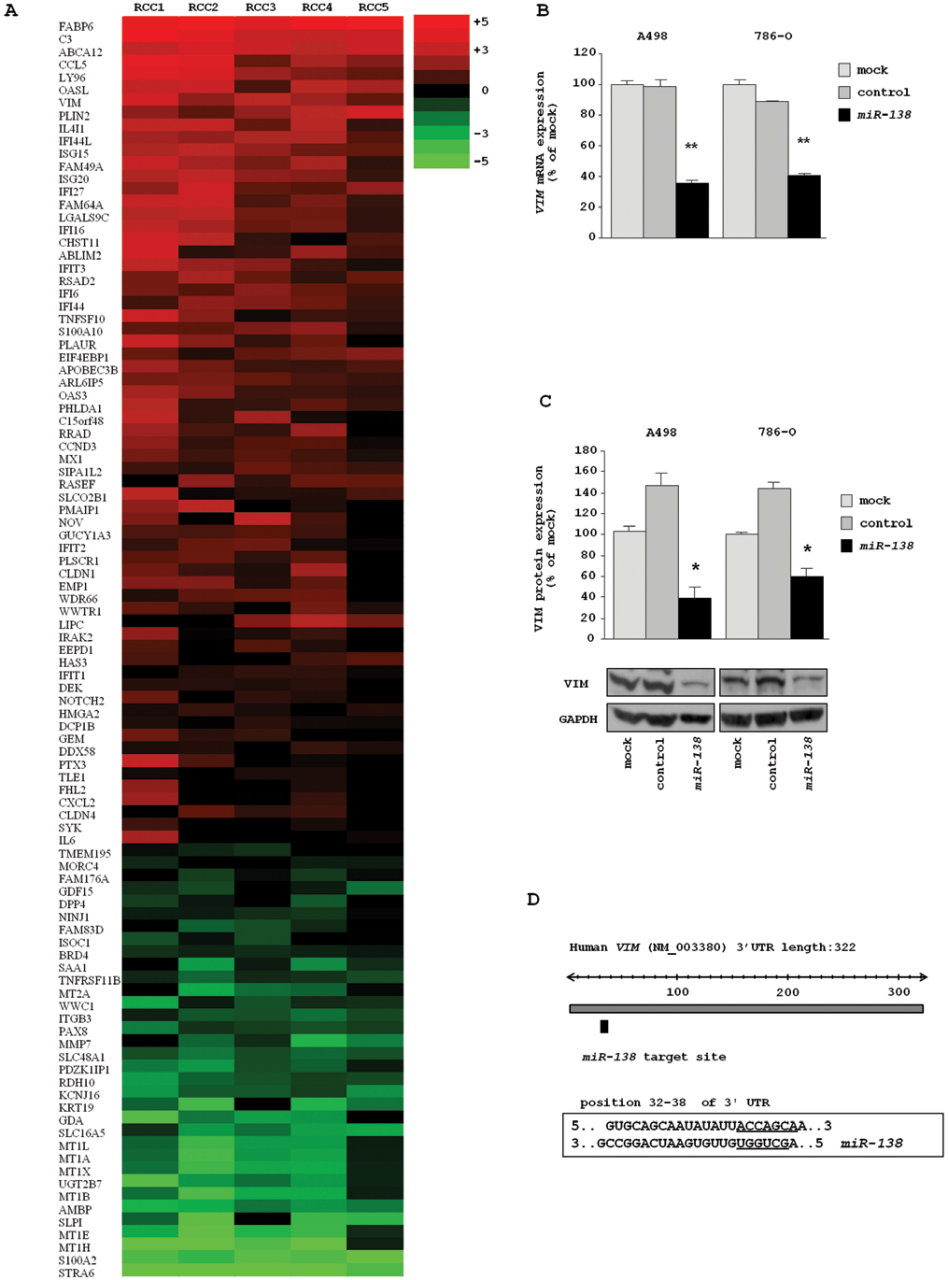
*miR-138* regulates molecular targets in RCC cells. (A) Heatmap derived from five RCC samples. A total of 99 genes were downregulated less than −2.0-fold in *miR-138* transfectants. We checked their mRNA expression levels in RCC by using our previous gene expression analysis of five RCC specimens. Ninety-nine gene expression levels are shown in the heat map diagram. *VIM* was the top upregulated gene among the genes which have miR-138 target sites in the heat map diagram. (B) *VIM* mRNA expression after 24 h transfection with 10 nM *miR-138*. (C) VIM protein expression after 72 h transfection of miRNAs. GAPDH was used as a loading control. The mRNA and protein levels of VIM were repressed in the transfectants. ^*^P<0.01, ^**^P<0.0001, (D) miR-138 binding sites in the 3′UTR of *VIM* mRNA.

Furthermore, we performed gene expression analysis using RCC clinical specimens (5 pairs of RCC and adjacent non-cancerous tissues). Several protein-coding genes were differentially expressed in this signature (data not shown). We selected 99 genes that were downregulated in *miR-138* transfectants and demonstrated their expression levels in a heatmap diagram (Fig. 2A). Entries from the microarray data were approved by the Gene Expression Omnibus (GEO), and were assigned GEO accession numbers GSE 36951 (RCC clinical specimens) and GSE 37119 (*miR-138* transfectants).

The two expression signatures in this study (*miR-138* transfectants and RCC clinical specimens) revealed that *VIM* was a promising putative target gene in *miR-138* in RCC. Thus, we focused on the *VIM* gene and investigated the functional significance of *VIM* in RCC cells.

### VIM as a direct target of repression by miR-138 in RCC cells

The mRNA and protein expression levels of VIM were markedly downregulated in *miR-138* transfectants (A498 and 768-O) in comparison with the mock and miRNA-control transfectants (Fig. 2B and C). The predicted target site of *miR-138* in *VIM* in the 3′UTR is shown in Fig. 2D.

### Silencing of VIM in RCC cell lines and the effect on cell proliferation, migration and invasion

**Figure 3:**
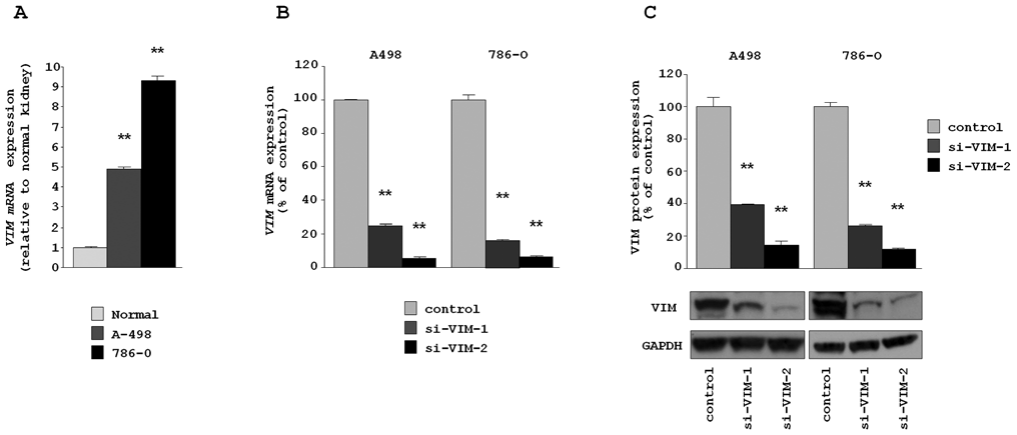
*VIM* expression was suppressed by si-*VIM* transfection on RCC cell lines. (A) The expression of *VIM* mRNAs in A 498 and 786-0 cell lines and normal kidney. The mRNA expression levels of *VIM* were 4- and 9-fold higher in RCC cell lines compared to the normal kidney RNA. *GUSB* was used as an internal control. (B) *VIM* mRNA expression after 24 h of transfection with 10 nM si-*VIM. VIM* mRNA expression was repressed in si-*VIM* transfectants. *GUSB* was used as an internal control. (C) VIM protein expression after 72 h transfection of si-*VIM*. GAPDH was used as a loading control. The expression level of VIM was also repressed in the transfectants.

First, we assessed the expression level of *VIM* in cells to be used for functional analysis of *VIM. VIM* mRNA expression levels in A498 and 786-O were significantly higher than those in normal human kidney RNA (relative to normal kidney RNA, 4.879±0.131 and 9.298±0.255, respectively, each P<0.0001) (Fig. 3A).

To examine the functional role of VIM, we performed loss-of-function studies using two different siRNAs, si-*VIM-1* and si-*VIM-2* transfected into A498 and 768-O cell lines. The mRNA and protein expression levels of VIM were markedly downregulated in both si-*VIM-1* and si-*VIM-2* transfectants (A498 and 768-O) in comparison with the siRNA-control transfectants (Fig. 3B and C). This result shows that two siRNA were useful for loss-of-function assays in this study.

**Figure 4:**
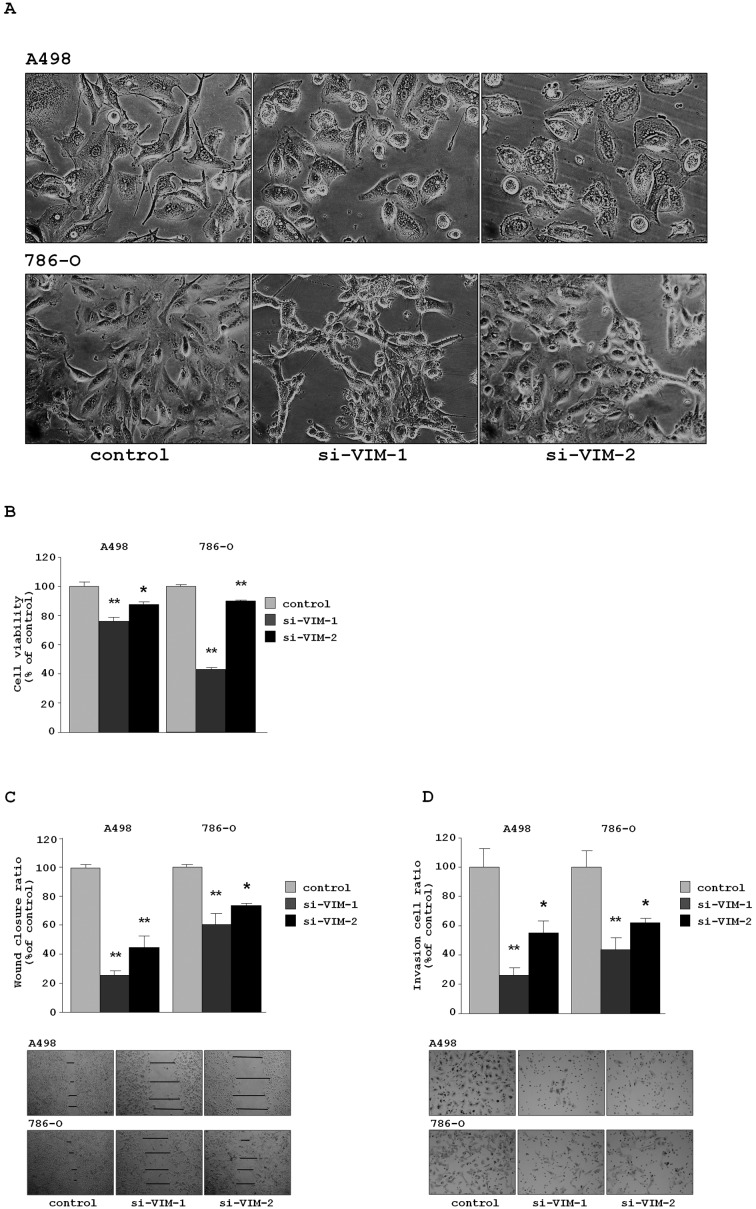
Response to *VIM* silencing by si-*VIM* transfection of RCC cell lines. (A) The change of morphology of si-*VIM* transfectants. A498 and 786-O cells were transfected with si-*VIM* for 72 h and were then examined by an inverted microscope. (B–D) VIM-knockdown effects on A498 and 786-0 cell lines transfected with si-*VIM-1* and si-*VIM-2*. (B) Cell proliferation determined by the XTT assay; (C) cell migration activity determined by the wound healing assay; and (D) cell invasion activity determined by the Matrigel invasion assay. ^*^P<0.005, ^**^P<0.0001.

Transfection of si-*VIM-1* and si-*VIM-2* in the RCC cell lines (A498 and 768-O) caused EMT-like changes in cell morphology, as that observed when cells were transfected with *miR-138* (Fig. 4A).

The XTT assay revealed that cell proliferation was inhibited in both si-*VIM*-transfectants in comparison with the si-control transfectants. The percentages of cell proliferation for A498 were 76.1±2.6, 87.5±1.9 and 100.0±3.1, respectively, P<0.0001 and P =0.0038. For 786-O, the values were 43.1±1.0, 89.9±0.7 and 100.0±1.1, respectively, P<0.0001 (Fig. 4B).

The wound healing assay demonstrated that significant inhibition of cell migration occurred in the si-*VIM*-transfectants in comparison with the si-control transfectants. The percentages of wound closure for A498 were 26.0±10.6, 44.7±8.4 and 100.0±2.6, respectively, each P<0.0001. For 786-O, the values were 60.8±7.3, 73.0±1.7 and 100.0±7.1, respectively, P<0.0001 and P=0.0002 (Fig. 4C).

The Matrigel invasion assay demonstrated that the number of invading cells significantly decreased in the si-*VIM*-transfectants in comparison with the si-control transfectants. For A498, the percentages of cells invading were 26.5±4.7, 54.8±8.3 and 100.0±12.4, respectively, P<0.0001 and P=0.0019. For 786-O, the values were 43.6±8.3, 61.6±3.3 and 100.0±11.1, respectively, P<0.0001 and P=0.0034 (Fig. 4D).

### Expression levels of miR-138 and VIM mRNA in RCC clinical specimens

**Figure 5:**
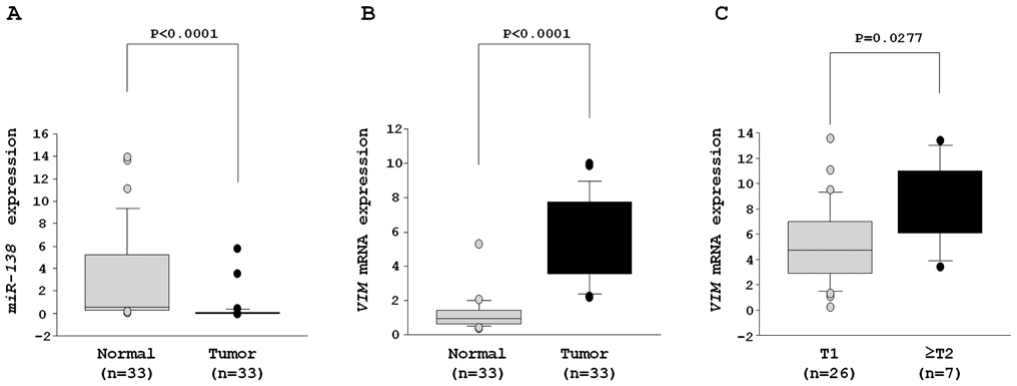
The expression levels of *miR-138* and *VIM* in RCC clinical specimens. (A and B) *miR-138* and *VIM* mRNA expression levels of 33 RCC and adjacent non-cancerous kidney tissues. Relative expression levels are expressed in box plots. (A) Expression levels of *miR-138* in RCC clinical samples were significantly downregulated compared with adjacent normal kidney. (B) Expression levels of *VIM* mRNA in RCC clinical samples were significantly upregulated compared with adjacent normal kidney samples. (C) The correlation of *VIM* mRNA between T1 and ≥T2 in RCC samples. *VIM* expression in ≥T2 RCC samples was significantly higher compared with T1 RCC samples.

Quantitative stem-loop RT-PCR demonstrated that the expression levels of *miR-138* were significantly reduced in 33 RCC samples (Table I) in comparison with adjacent non-cancerous specimens (clinical RCC specimens, 0.346±0.201 versus adjacent normal tissues, 2.983±0.715, P<0.0001) (Fig. 5A).

On the other hand, the mRNA expression level of *VIM* was significantly higher in RCC than adjacent non-cancerous specimens (clinical RCC specimens, 6.017±0.622, adjacent normal tissues; 1.316±0.224, P<0.0001) (Fig. 5B).

The mRNA expression of ≥T2 specimens (n=7) was significantly higher than that of T1 (n=26) (T1, 5.230±0.607; ≥T2, 8.773±1.349, P=0.0277) (Fig. 5C).

### Immunohistochemistry of VIM in tissue microarray

**Figure 6:**
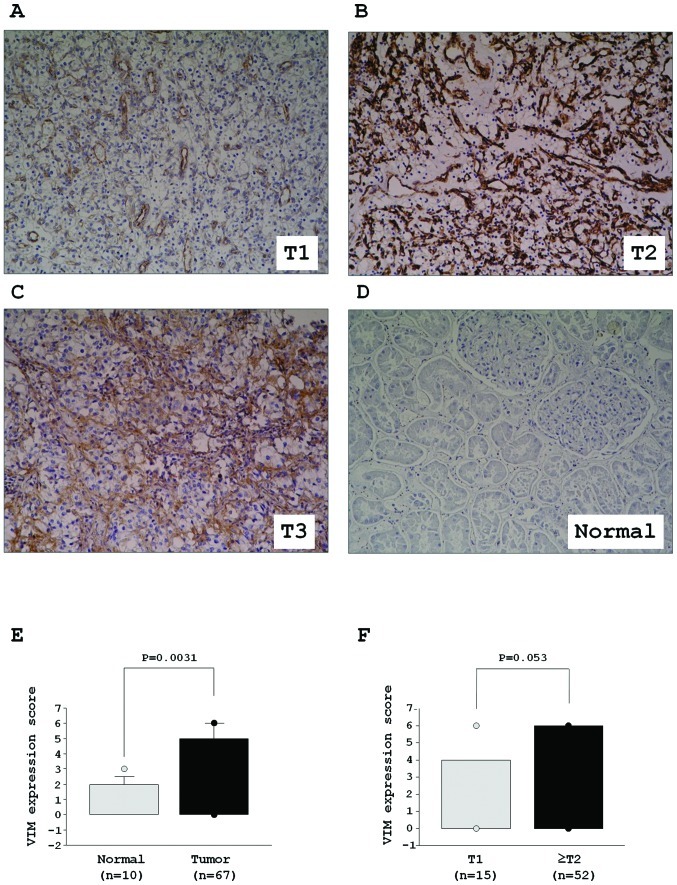
Immunohistochemical staining of VIM in tissue microarray. (A–C) Positively stained tumor lesions (A) T1 N0 M0; (B) T2 N0 M0 and (C) T3 N0 M0. (D) Negative staining in normal kidney tissue. (E–F) VIM expression levels in immunohistochemical staining; (E) VIM expression in normal kidney and RCC; (F) correlation between VIM expression and clinic pathologic parameters in RCC.

VIM was detected by immunohistochemical staining. Fig. 6A–D shows representative results of immunohistochemical staining of VIM. VIM was strongly expressed in tumor lesions (Fig. 6A–C), whereas no expression was observed in normal tissue (Fig. 6D). The expression score of VIM was significantly higher in 67 RCC specimens in comparison with ten normal kidney specimens (Fig. 6E). The VIM expression of ≥T2 specimens (n=52) was higher than that of T1 (n=15). There was a trend but no significant difference in the expression score of VIM between T1 and ≥T2 specimens (P=0.053, Fig. 6F). While there was a trend between VIM expression and grade, the difference was not significant. Patient characteristics are summarized in Table III.

## Discussion

The incidence of RCC has increased over the last few decades, and it currently represents approximately 2% of all cancer-related deaths (25). Although two-thirds of RCC patients have clinically localized disease and will undergo curative surgery, up to 40% of patients develop distant metastasis and their outcomes are poor (2–4). Many studies have indicated that cell adhesion and extra-cellular matrix proteins contribute to the cells’ acquired abilities for invasion, migration and metastasis (26).

EMT is an embryologically conserved genetic program that is an essential step enabling cancer cell invasion and metastasis. For example, epithelial cells lose intercellular tight junctions and polarity (27). Recent data indicate that the *microRNA-200* family (*miR-200a, -200b, -200c, -141* and *-429*) is downregulated in aggressive human cancers. Moreover, it plays critical roles in the inhibition of key regulators of EMT and β-catenin/Wnt signaling (28,29). Interestingly, our miRNA expression signature of RCC showed that all *miR-200* family members were reduced in clinical specimens and that restoration of the *miR-200* family inhibited cancer cell migration and invasion (data not shown).

Our previous study showed that *miR-138* was reduced in RCC miRNA expression signature (12), we validated the down-regulation of *miR-138* in RCC clinical specimens in this study. Aberrant expression of *miR-138* has been observed in several types of cancer such as head and neck squamous cell carcinoma (HNSCC) (30), anaplastic thyroid carcinoma (19) and lung cancer (20). Two *miR-138* precursor genes, *miR-138-1* and *miR-138-2*, have identical sequences in the mature miRNA and map to human chromosomes 3p21.33 and 16q13, respectively. Although it is believed that genomic deletion or epigenetic silencing of miRNA in cancer cells, the molecular mechanism of downregulated miRNAs in RCC is not clear. In the human chromosomal region 3p, LOH is frequently observed in many cancers including RCC (31,32). This problem can be solved by genome-based high throughput analysis in each clinical case.

Importantly, we found significant morphologic change in RCC cell lines (A498 and 786-O) by *miR-138* transfection. Furthermore, restoration of *miR-138* significantly inhibited cancer cell migration and invasion in RCC cells. These data suggested that *miR-138* functions as a tumor suppressor that inhibits RCC invasion and metastasis. miRNAs are unique in their ability to regulate many protein-coding genes. A single miRNA is capable of targeting a number of genes to regulate biological processes globally. Bioinformatic predictions suggest that miRNAs regulate more than 30% of protein coding genes (9). The elucidation of new molecular pathways regulated by tumor suppressive *miR-138* is important for our understanding of human RCC invasion and metastasis. Based on this view, we performed molecular target searches for *miR-138* in cancer cells by combining two genome-wide gene expression studies (*miR-138* transfectants and RCC mRNA clinical signature) and *in silico* analysis.

In this study, we focused on *VIM* as a putative candidate of *miR-138* in RCC cells. We chose *VIM* for the following reasons. First, downregulation of *VIM* was recognized in the expression signature of *miR-138* transfectants. Second, overexpression of *VIM* was observed in RCC clinical specimens. Third, *VIM* has a putative *miR-138* target site in its 3′ untranslated region. Our data demonstrated that restoration of *miR-138* significantly inhibited both mRNA and protein expression levels of *VIM* in RCC cells, suggesting *VIM* was regulated by tumor suppressive *miR-138*. It is well known that VIM is an essential constituent of cytoskeletal proteins of mesenchymal cells and VIM is a marker of EMT (21). During EMT, cytoskeletal proteins are changed from keratin-rich networks to VIM-rich networks connected to focal adhesions. Morphological changes of RCC cells and accelerated cell migration and invasion is caused by the reduction of *miR-138* and the upregulation of *VIM* pathways. Interestingly, it has been shown that *miR-138* regulated cell migration and invasion by targeting *RhoC*, *ROCK*, *ZEB2*, *EZH2* and *VIM* in HNSCC cells (33,34). Importantly, restoration of *miR-138* in an HNSCC cell line changed the EMT-like cell morphology and suppressed cell migration and invasion (34). This result is in accord with the data obtained in our study of RCC. Furthermore, overexpression of *miR-138* reduced cell viability and colony formation in HCC cell lines targeting *CCND3*(35). The report also showed that protein expression of CCND3 was negatively correlated with *miR-138* expression in HCC tissues. Our data of *miR-138* transfectants in RCC cell lines demonstrated that *CCND3* is a putative target of *miR-138* in RCC, suggesting that *miR-138* regulation of the *CCND3* pathway is important for RCC oncogenesis.

The results of this study and previous data indicate that *VIM* is a functional target of tumor suppressive *miR-138*, and this pathway contributes to cancer cell migration, invasion, and metastasis. In this study, we also demonstrated overexpression of VIM in clinical specimens of RCC. Previous studies indicated that VIM was a sensitive and specific marker for conventional RCCs (36,37). The combination of VIM and CD9 staining was found to distinguish clear cell RCC and chromophobe RCC (37). Our tissue microarray data showed a positive correlation between VIM expression and tumor grade in RCC specimens. In this analysis, we were not able to obtain a correlation of *VIM* expression and metastasis in RCC patients. Silencing of *VIM* in RCC cell lines changed cell morphology and significantly inhibited cell migration and invasion in this study. Thus, we propose that overexpression of *VIM* participates in metastasis of RCC. Studies of a large number of samples with balanced pathological backgrounds are needed to elucidate the precise correlation between *VIM* and/or *miR-138* expression and clinicopathological parameters.

In summary, the reduction of *miR-138* and the increased expression of *VIM* are frequent events in RCC clinical specimens. Restoration of *miR-138* in RCC cells changed the EMT-like morphology and suppressed cell migration and invasion. The tumor suppressive *miR-138*-mediated cancer pathway provides new insights into the potential mechanisms of RCC oncogenesis and metastasis.

## Figures and Tables

**Table I:** Patient characteristics of RT-PCR experiments.

	No. of patients (%)	
Total number	33	
Age (average)	36–83	(65.6)
Gender		
Male	22	(66.7)
Female	11	(33.3)
Pathological tumor stage		
pT1a	12	(36.4)
pT1b	14	(42.4)
pT2	2	(6.1)
pT3a	3	(9.1)
pT3b	2	(6.1)
pT4	0	(0.0)
Grade		
G1	5	(15.2)
G2	26	(78.8)
G3	0	(0.0)
Unknown	2	(6.1)
Infiltration		
α	12	(36.4)
β	21	(63.6)
γ	0	(0.0)
Venous invasion		
v (−)	24	(72.7)
v (+)	9	(27.3)

**Table II:** Downregulated genes in *microRNA-138* transfectants.

Entrez gene ID	Symbol	Average	Target site
3569	IL6	−5.35 interleukin 6 (interferon, β 2)	(−)
4856	NOV	−5.22 nephroblastoma overexpressed gene	(−)
84448	ABLIM2	−4.97 actin binding LIM protein family, member 2	(−)
3773	KCNJ16	−4.86 potassium inwardly-rectifying channel, subfamily J, member 16	(−)
6352	CCL5	−4.44 chemokine (C-C motif) ligand 5	(−)
4316	MMP7	−4.1 matrix metallopeptidase 7 (matrilysin, uterine)	(−)
3038	HAS3	−4.03 hyaluronan synthase 3	(+)
91543	RSAD2	−3.99 radical S-adenosyl methionine domain containing 2	(−)
5806	PTX3	−3.85 pentraxin-related gene, rapidly induced by IL-1 β	(−)
64220	STRA6	−3.85 stimulated by retinoic acid gene 6 homolog (mouse)	(+)
84419	C15orf48	−3.8 chromosome 15 open reading frame 48	(−)
144406	WDR66	−3.67 WD repeat domain 66	(−)
4493	MT1E	−3.66 metallothionein 1E	(−)
718	C3	−3.65 complement component 3	(−)
10964	IFI44L	−3.64 interferon-induced protein 44-like	(−)
3990	LIPC	−3.64 lipase, hepatic	(−)
9121	SLC16A5	−3.61 solute carrier family 16, member 5 (monocarboxylic acid transporter 6)	(−)
4490	MT1B	−3.6 metallothionein 1B	(−)
8091	HMGA2	−3.56 high mobility group AT-hook 2	(−)
1803	DPP4	−3.49 dipeptidyl-peptidase 4	(−)
6288	SAA1	−3.48 serum amyloid A1	(−)
4502	MT2A	−3.44 metallothionein 2A	(−)
8638	OASL	−3.43 2′-5′-oligoadenylate synthetase-like	(−)
9582	APOBEC3B	−3.39 apolipoprotein B mRNA editing enzyme, catalytic polypeptide-like 3B	(−)
4500	MT1L	−3.32 metallothionein 1L (gene/pseudogene)	(−)
3437	IFIT3	−3.3 interferon-induced protein with tetratricopeptide repeats 3	(−)
9076	CLDN1	−3.05 claudin 1	(−)
8743	TNFSF10	−3.03 tumor necrosis factor (ligand) superfamily, member 10	(−)
3433	IFIT2	−2.94 interferon-induced protein with tetratricopeptide repeats 2	(−)
2172	FABP6	−2.91 fatty acid binding protein 6, ileal	(−)
23586	DDX58	−2.89 DEAD (Asp-Glu-Ala-Asp) box polypeptide 58	(−)
4982	TNFRSF11B	−2.89 tumor necrosis factor receptor superfamily, member 11b	(−)
259307	IL4I1	−2.88 interleukin 4 induced 1	(−)
6590	SLPI	−2.88 secretory leukocyte peptidase inhibitor	(−)
5174	PDZK1	−2.88 PDZ domain containing 1	(−)
51015	ISOC1	−2.86 isochorismatase domain containing 1	(+)
3434	IFIT1	−2.84 interferon-induced protein with tetratricopeptide repeats 1	(−)
22822	PHLDA1	−2.79 pleckstrin homology-like domain, family A, member 1	(−)
2537	IFI6	−2.76 interferon, α-inducible protein 6	(−)
392636	TMEM195	−2.76 transmembrane protein 195	(−)
81610	FAM83D	−2.73 family with sequence similarity 83, member D	(+)
26154	ABCA12	−2.66 ATP-binding cassette, sub-family A (ABC1), member 12	(−)
4940	OAS3	−2.65 2′-5′-oligoadenylate synthetase 3, 100 kDa	(+)
5359	PLSCR1	−2.65 phospholipid scramblase 1	(−)
6236	RRAD	−2.61 Ras-related associated with diabetes	(−)
4496	MT1H	−2.56 metallothionein 1H	(−)
4814	NINJ1	−2.54 ninjurin 1	(+)
11309	SLCO2B1	−2.5 solute carrier organic anion transporter family, member 2B1	(+)
158158	RASEF	−2.47 RAS and EF-hand domain containing	(−)
259	AMBP	−2.47 α-1-microglobulin/bikunin precursor	(−)
2669	GEM	−2.47 GTP binding protein overexpressed in skeletal muscle	(−)
3656	IRAK2	−2.42 interleukin-1 receptor-associated kinase 2	(−)
3880	KRT19	−2.41 keratin 19	(−)
1978	EIF4EBP1	−2.41 eukaryotic translation initiation factor 4E binding protein 1	(+)
7431	VIM	−2.39 vimentin	(+)
57568	SIPA1L2	−2.38 signal-induced proliferation-associated 1 like 2	(−)
7913	DEK	−2.35 DEK oncogene	(+)
123	PLIN2	−2.34 perilipin 2	(−)
4501	MT1X	−2.33 metallothionein 1X	(−)
654346	LGALS9C	−2.31 lectin, galactoside-binding, soluble, 9C	(+)
4489	MT1A	−2.3 metallothionein 1A	(−)
3669	ISG20	−2.28 interferon stimulated exonuclease gene 20 kDa	(−)
2920	CXCL2	−2.28 chemokine (C-X-C motif) ligand 2	(−)
2274	FHL2	−2.27 four and a half LIM domains 2	(−)
157506	RDH10	−2.27 retinol dehydrogenase 10 (all-trans)	(−)
25937	WWTR1	−2.26 WW domain containing transcription regulator 1	(−)
3690	ITGB3	−2.26 integrin, β 3 (platelet glycoprotein IIIa, antigen CD61)	(+)
196513	DCP1B	−2.24 DCP1 decapping enzyme homolog B (*S. cerevisiae*)	(−)
9518	GDF15	−2.24 growth differentiation factor 15	(−)
1364	CLDN4	−2.23 claudin 4	(−)
23643	LY96	−2.2 lymphocyte antigen 96	(−)
10561	IFI44	−2.2 interferon-induced protein 44	(−)
84141	FAM176A	−2.19 family with sequence similarity 176, member A	(−)
6281	S100A10	−2.17 S100 calcium binding protein A10	(−)
7088	TLE1	−2.17 transducin-like enhancer of split 1 (E(sp1) homolog, *Drosophila*)	(−)
81553	FAM49A	−2.17 family with sequence similarity 49, member A	(−)
4599	MX1	−2.17 myxovirus (influenza virus) resistance 1, interferon-inducible protein p78 (mouse)	(−)
6850	SYK	−2.17 spleen tyrosine kinase	(−)
7364	UGT2B7	−2.17 UDP glucuronosyltransferase 2 family, polypeptide B7	(−)
5366	PMAIP1	−2.17 phorbol-12-myristate-13-acetate-induced protein 1	(−)
50515	CHST11	−2.16 carbohydrate (chondroitin 4) sulfotransferase 11	(+)
2982	GUCY1A3	−2.16 guanylate cyclase 1, soluble, α 3	(+)
6273	S100A2	−2.15 S100 calcium binding protein A2	(+)
54478	FAM64A	−2.15 family with sequence similarity 64, member A	(−)
3428	IFI16	−2.14 interferon, γ-inducible protein 16	(−)
9615	GDA	−2.14 guanine deaminase	(−)
7849	PAX8	−2.13 paired box 8	(−)
10550	ARL6IP5	−2.11 ADP-ribosylation-like factor 6 interacting protein 5	(+)
23286	WWC1	−2.1 WW and C2 domain containing 1	(+)
9636	ISG15	−2.08 ISG15 ubiquitin-like modifier	(+)
896	CCND3	−2.07 cyclin D3	(+)
5329	PLAUR	−2.07 plasminogen activator, urokinase receptor	(−)
4853	NOTCH2	−2.06 Notch homolog 2 (*Drosophila*)	(+)
55652	SLC48A1	−2.05 solute carrier family 48 (heme transporter), member 1	(+)
23476	BRD4	−2.04 bromodomain containing 4	(+)
2012	EMP1	−2.03 epithelial membrane protein 1	(+)
3429	IFI27	−2.02 interferon, α-inducible protein 27	(−)
79710	MORC4	−2.01 MORC family CW-type zinc finger 4	(−)
80820	EEPD1	−2.01 endonuclease/exonuclease/phosphatase family domain containing 1	(+)

**Table III:** Patient characteristics of immunohistochemistry.

	No. of patients (%)	
Total number	67	
Age (average)	30–80	(54.4)
Gender		
Male	45	(67.2)
Female	22	(32.8)
Pathological tumor stage		
pT1	15	(22.4)
pT2	28	(41.8)
pT3	22	(32.8)
pT4	2	(3.0)
Grade		
G1	52	(77.6)
G2	14	(20.9)
G3	1	(1.5)
Normal tissue	10	
